# Rotavirus Vaccine: A Promise for the Future

**DOI:** 10.3329/jhpn.v26i4.1879

**Published:** 2008-12

**Authors:** K. Zaman

**Affiliations:** 1 Scientist and Epidemiologist Child Health Unit Public Health Sciences Division ICDDR, B GPO Box 128, Dhaka 1000 Bangladesh

Rotaviruses—non-enveloped, double-stranded RNA viruses—infect both humans and animals and are distributed worldwide. In humans, rotavirus causes diarrhoea of varying severity ranging from mild to severe. Symptoms, such as diarrhoea, vomiting, and fever, begin after an incubation period of 2-3 days and persist, on average, for six days (1). Rotavirus is the leading cause of severe diarrhoea in infants and young children, accounting for 45% of severe diarrhoeal disease in both developed and developing countries (2,3), responsible for ~5% of all deaths and 16% of potentially vaccine-preventable deaths among children globally (2,4). Rotaviruses are associated with 55% of all diarrhoea-related hospitalizations among children aged less than five years as revealed in the reports of the Asian Rotavirus Surveillance Network (5). Virtually, all children throughout the world are infected with rotavirus by the time they are 3-5 years old, regardless of the socioeconomic status or environmental conditions (2,6).

The World Health Organization has recently esti-mated that 611,000 (range 454,000-705,000) deaths due to rotavirus occur in children aged less than five years, accounting for approximately 20-25% of all deaths due to diarrhoeal disease (7). Of the deaths due to rotavirus occurring worldwide, it is estimated that approximately 145,000 deaths occur in sub-Saharan Africa and approximately 200,000 deaths occur in Asia (8-10).

Studies have shown that rotavirus-associated disease occurs round the year in tropical climates with no consistent seasonal variation, although there may be more peaks in the dry, cool months, or winter months (11). Serotype G1, G2, G3, and G4 are responsible for 88% of gastroenteritis due to rotavirus, worldwide (12). These serotypes also account for over 80% of strains in Africa and over 90% of strains in Asia. Other G serotypes that have become more common over the last decade include G9 (globally) and G8 (in Africa). Unusual G and P type combinations have been identified in Africa with an increasing frequency (13).

Safe and effective rotavirus vaccines are needed to reduce the enormous public-health burden associated with illness due to rotavirus, especially in developing countries (14). The large global burden of healthcare due to rotavirus-associated disease in both developed and developing countries prompted the development of rotavirus vaccines. Prevention by vaccination is considered to be critical for effective control of infection due to rotavirus since it cannot be prevented with improvements in water and sanitation (11), and only non-specific symptomatic therapies are available. Various approaches to the development of rotavirus vaccines have been undertaken, with live-attenuated oral vaccines receiving the most attention.

The first licensed rotavirus vaccine—RotaShield—a tetravalent rhesus rotavirus vaccine was manufactured by Wyeth-Lederle and was licensed in the USA in 1998, and marketing authorization was granted for Europe in 1999 but was withdrawn from the market in 1999 due to an increased risk of intussusception shortly after its administration (15,16). Unanticipated adverse events (intussusception) experienced with the RotaSheild vaccine have accelerated efforts to develop and evaluate alternative vaccine candidates so that a safe and effective public-health tool would become available. Two new rotavirus vaccines—Rotarix by GSK and Rotateq by Merck—have been developed. Both the vaccines were tested in both industrialized and middle-income countries among more than 60,000 infants which demonstrated its safety and efficacy. The vaccine had an efficacy of 85-98% against severe rotavirus-associated disease requiring hospitalization and reduced all-cause hospitalizations by 42-59%, preventing nearly all hospitalizations for rotavirus (17,18). Both the vaccines did not interfere with the immunogenicity when co-administered with other EPI vaccines, including polio (19-21). The vaccines were not associated with an increased risk of intussusception.

This issue of the Journal includes an article by Cons-tenla *et al*. who evaluated the cost-effectiveness of a national rotavirus-vaccination progrmme in Brazilian children (22). The authors developed a model to estimate the disease outcomes and healthcare costs associated with diarrhoea due to rotavirus in a hypothetical annual birth-cohort of children for a five-year period. It has been estimated that rotavirus vaccination in Brazil would prevent more than three-fourths of outpatient visits, hospitalizations, and deaths. The authors conclude that the rotavirus-vaccination programme would be cost-effective and would provide an effective opportunity for improving child health in Brazil.

Further analyses were done to estimate the economic burden and cost-effectiveness of rotavirus vaccination in Asia. The universal vaccination programme in Asia would also reduce a substantial proportion of the burden of rotavirus-associated disease and related healthcare costs in Asia (23). They estimated reduction of 109,000 deaths (64%), 1.4 million hospitalizations (74%), 7.7 million outpatient visits (57%), and US$ 139 million (73%) in healthcare costs.

Both the vaccines—Rotarix and Rotateq—were approved by the FDA, and the vaccines have been licensed in many countries. Different countries have already adopted the rotavirus vaccine in their routine EPI programme. Post-licensure monitoring of the burden of rotavirus-associated disease, safety of vaccine, and determination of circulating rotavirus strains are needed. However, several challenges remain before the widespread use of rotavirus vaccines in developing countries. These include price-related issues, awareness of diseases due to rotavirus, and acceptance of the vaccine (24,25).

There are concerns about the efficacy of rotavirus vaccine in developing countries since live oral enteric vaccines (polio, rotavirus) have been shown to be less immunogenic compared to developed countries (26,27; Steele AD. Personal communication, 2008). Further research would be helpful to determine the safety and efficacy of the vaccines, particularly among children in developing countries with a high prevalence of malnutrition, widespread circulation of intestinal micro-organisms, and accompanying diseases, such as malaria and HIV. The demonstration of immune responses in presence of high maternal antibodies and breastfeeding at the time of vaccination is important. The results of the ongoing rotavirus efficacy trials in Asia (Bangladesh and Viet Nam) and Africa (Kenya, Ghana, and Mali) would provide information whether the vaccine works well in developing countries. The rotavirus-effectiveness study which has just been started in Bangladesh will provide experience with the vaccine in a ‘real-world’ setting, and lessons will be learnt on vaccine delivery in the villages of Bangladesh. This would be helpful for the health ministry to decide to include the vaccine in its EPI programme for a rapid scale-up.

The inclusion of rotavirus vaccine in the routine childhood-immunization programme in developing countries will help achieve the Millennium Development Goal of reducing childhood mortality through prevention of deaths due to rotavirus-associated diseases (28).
